# Book Review: Sex/Gender

**DOI:** 10.3389/fpsyg.2020.573606

**Published:** 2020-11-16

**Authors:** Xiufeng Zhang, Hengwen Yang

**Affiliations:** ^1^School of Foreign Languages, Jiujiang University, Jiujiang, China; ^2^Department of English Linguistics and Literature, Dong-A University, Busan, South Korea

**Keywords:** gender studies, male study, queer theory, feminism, China

*Sex/Gender* is 248 pages in total and is authored by Liu Yan, who holds a Ph.D. in literary studies from The Chinese University of Hong Kong which was awarded in 2003. She is currently working as a professor of English at Faculty of English Language and Culture, Guangdong University of Foreign Studies (Guangzhou, P.R. China). Her academic interests include psychoanalysis and gender studies. This book makes a necessary contribution to the gap in Chinese scholarship regarding male and LGBTQ studies which are often overshadowed by more Feminist studies (women's studies) in Chinese academic circles.

This book analyzes three major branches of gender studies and specific case studies with the aim to explain, shape, and broaden the direction of further research by Chinese academics in this field. Women's, male, and LGBTQ studies are discussed from both Western and Chinese perspectives enabling the reader to contrast and grasp the contextual differences inherent in both. Starting from a point of cross-culturally analyzing the differences between sex and gender and its evolution, the author(s) uses this as a foundation to discuss women's, male, and LGBTQ gender studies and draws on a number of English language literary case studies to establish a path into how Western theories of gender-related literary criticism may be crafted into something new and relevant to the Chinese context.

The five chapters of the book can be grouped into four parts. Part 1 (chapter one) analyses the evolution and differences between sex and gender both from a Chinese context and an English cultural perspective. It establishes a thorough understanding of the sex/gender distinction, current debate into the distinction, and establishes its significance for gender studies in China as well as a general introduction into Chinese gender studies. According to Liu Yan et al., sex is a fixed biological category assigned at birth, while gender is a spectrum of sex constructed by the social and cultural role of each sex and extends the boundary beyond binary male and female to LGBTQ. Sexuality denotes to a person's sexual attraction or orientation to a particular sex which could divide into four categories: *heterosexuality*; *homosexuality*; *bisexuality*, and *asexuality*.

Part 2 (chapter two) discusses the major themes of women's, male, and LGBTQ (lesbian, gay, bisexual, transgender, and queer) studies under the umbrella of gender studies as a whole. The author's treatment of women's studies focuses on feminine subjectivity, the female body, and feminist writing. These core concepts enable the readers to construct a theoretical base for understanding feminism theory. The author(s) look at the study of men through the lens of masculinity, male identity, and male violence. The study of masculinity in Chinese academic circles mainly focuses on the fields of sociology and pedagogy and centers on how masculinity is formed and reinforced, its reproduction in literary culture, and the different types of masculinity encountered among different male groups. Significantly, research into Chinese masculinity only began in the late twentieth century as a new offshoot after Chinese women's studies were firmly established. The study into LGBTQ themes covers other types of gendered individuals and groups and reflects on how social norms have affected them and tends to break the dualistic choice between coming out and remaining closeted.

Part 3 (chapter three and chapter four) aims to make readers better understand the abovementioned three theories in gender studies through a variety of case studies. These case studies enable readers to understand these theories with specific literary case studies. Contained within these case studies is a working blueprint for how similar studies should be conducted and it forms a methodological guide for readers who wish to conduct similar studies. For example, Feminist analysis is focused on the “Female Subjectivity of Knowledge in Cynthia Ozick's Novel” to explore the relation between knowledge and female subjectivity. It is crucial for women to obtain knowledge and then construct female identity in a patriarchy society.

Part 4 (Chapter five) reviews previous gender studies based on literary criticism and looks at the new trends in gender studies in China. It is here that the authors posit that even though gender studies emerged from western history, it is better for Chinese scholars to consider how to use these western theories to do research on local phenomena. The authors put forward localization strategies for the development of gender studies especially feminism in China. First of all, Chinese scholars should adopt a global perspective to Chinese gender studies. Secondly, it is better to draw on the research results of overseas Chinese scholars and sinologists. Finally, it is important to employ a “close reading” strategy in literary and cultural criticism analysis and avoid applying the Western gender theories mechanically.

Furthermore, the authors propose some new perspectives for future gender studies in China. Firstly, intersectionality theory would offer a new perspective such as *class, region, and race* for gender studies in China. Secondly, they contend that a multidisciplinary analysis of sexuality will emerge as a new trend in gender studies. Finally, gender studies should move toward bio-politics research which cares for vulnerable groups in gender such as those with disabilities, the elderly, children, and other groups.

The contribution this book makes to the study of Chinese gender studies is noteworthy and possibly far-reaching. It combines gender studies with specific case studies so that readers can grasp the development stages of gender studies in China theoretically and understand how to apply these theories to analyze literary works. Another noteworthy contribution is that Liu rightly reminds the reader of the importance and necessity of developing more original work based on localized gender studies frameworks developed in China and the need to move away from just translating western theories into Chinese (without considering the Chinese context). A third contribution of the reviewed book lies in that it predicts the future trends of gender studies in China. Future gender studies according to the author(s) will focus on those marginalized groups in gender such as those with disabilities, the elderly, children, and other groups from a multidisciplinary and intersectionality perspective. Therefore, it is these qualities that make *Sex/Gender* a good book for post-graduate students, researchers, and scholars interested in gender studies, English literature, and sociology studies.

To conclude, what makes this book distinctive from other research is that Liu Yan extends her research from feminist criticism to male research and LGBTQ studies from a Chinese perspective. This book adds more theoretical explanations and also provides a practical research method to guide future gender studies in China.

However, there are some limitations in the book. Although the book points out and recommends new ways to avoid mechanically overreliance on Western gender principles and methodology to Chinese gender studies, the book did not provide any specific Chinese examples. In particular, the six case studies are all borrowed from western literary works. Therefore, more studies could be done to draw on western perspectives and appropriately applied to a Chinese context and the study of bio-politics from a multidisciplinary and intersectionality perspective.

## Author Contributions

XZ has written the book review. HY helped to examine and modify the review. Both authors contributed to the article and approved the submitted version.

## Conflict of Interest

The authors declare that the research was conducted in the absence of any commercial or financial relationships that could be construed as a potential conflict of interest.

